# *De Novo* Transcriptome Assembly and Identification of Gene Candidates for Rapid Evolution of Soil Al Tolerance in *Anthoxanthum odoratum* at the Long-Term Park Grass Experiment

**DOI:** 10.1371/journal.pone.0124424

**Published:** 2015-07-06

**Authors:** Billie Gould, Susan McCouch, Monica Geber

**Affiliations:** 1 Department of Plant Biology, Plant Biology Laboratories, Michigan State University, East Lansing, MI 48824, United States of America; 2 Department of Ecology and Evolutionary Biology, Cornell University, Corson Hall Tower Rd, Ithaca, NY 14853, United States of America; 3 Department of Plant Breeding and Genetics, Cornell University, Emerson Hall Tower Rd, Ithaca, NY 14853, United States of America; Institute for Sustainable Agriculture (IAS-CSIC), SPAIN

## Abstract

Studies of adaptation in the wild grass *Anthoxanthum odoratum* at the Park Grass Experiment (PGE) provided one of the earliest examples of rapid evolution in plants. *Anthoxanthum* has become locally adapted to differences in soil Al toxicity, which have developed there due to soil acidification from long-term experimental fertilizer treatments. In this study, we used transcriptome sequencing to identify Al stress responsive genes in *Anthoxanhum* and identify candidates among them for further molecular study of rapid Al tolerance evolution at the PGE. We examined the Al content of *Anthoxanthum* tissues and conducted RNA-sequencing of root tips, the primary site of Al induced damage. We found that despite its high tolerance *Anthoxanthum* is not an Al accumulating species. Genes similar to those involved in organic acid exudation (*TaALMT1*, *ZmMATE)*, cell wall modification (*OsSTAR1*), and internal Al detoxification (*OsNRAT1*) in cultivated grasses were responsive to Al exposure. Expression of a large suite of novel loci was also triggered by early exposure to Al stress in roots. Three-hundred forty five transcripts were significantly more up- or down-regulated in tolerant vs. sensitive *Anthoxanthum* genotypes, providing important targets for future study of rapid evolution at the PGE.

## Introduction

The Park Grass Experiment (PGE, Harpenden, UK) is a unique site where both radical changes in plant species composition and rapid evolution within populations have been documented in response to experimental soil manipulations over the past 150+ years. The experiment began in 1856 to test the effects of soil management practices on haymeadow productivity. Within a large sub-divided field, experimental fertilizer applications have caused the soil to acidify on some plots, and liming treatments applied on a subset of plots has counteracted the acidification process [1]. Sweet vernal grass, *Anthoxanthum odoratum*, is one of only a few species that grow across plots of almost all acidity levels at PGE. It is an outcrossing, tetraploid grass (2n = 20) native to Europe, that reproduces mainly by seed and has been the focus of several studies of trait differentiation in response to rapid soil nutrient shifts [2–7]. Davies and Snaydon (1973) [8] demonstrated that *A*. *odoratum* growing at the PGE has evolved locally adaptive differences in tolerance to soil aluminum (Al), which becomes highly toxic under acid soil conditions. In a recent study, we observed the same pattern of locally adaptive differences in Al tolerance in both adult plants and seedlings across a sample of 108 genotypes from 8 of the most and least acidic soil subplots at the PGE [9]. Others have shown using neutral genetic markers that the subpopulations are only weakly differentiated from each other and adaptive differences have evolved despite a high rate of gene flow [1,10,11]. Since the initial tests of local adaptation to Al toxicity at the PGE, work on domesticated plants has yielded detailed molecular information on the basis of soil Al tolerance in domesticated grasses such as rice, wheat, sorghum and corn. This information provides a useful comparative resource for exploring the genetic basis of Al tolerance in wild grasses such as *Anthoxanthum*.

Soil Al toxicity presents a challenge for plants in both agriculturally managed and natural acid soils worldwide. Al is the third most abundant element in the earth’s crust and is present in most soils, however under acid conditions (pH<5.0) is present mostly as mononuclear cations (Al^3+^) that are phytotoxic to most herbaceous plants even at low concentration [12]. The primary way Al^3+^ causes damage is by attaching to root cell walls, causing them to become rigid and form lesions as they expand through soil [13]. Most cultivated plants have low to moderate Al tolerance and production is severely limited in acid soils. For this reason, tolerance has been studied intensively in grasses such as sorghum, rice, wheat, and maize [14–16]. It is well known, however, that some wild plants surpass crop species in their level of Al tolerance [17–21]. In these species almost nothing is known about the genetic basis of the trait.

At a coarse level, plants can be split into two groups with regard to Al physiology: 1) those that are highly tolerant and specialized for growth on acid soils by way of an ability to sequester large amounts of Al in non-toxic forms in their tissues (Al accumulators), and 2) plants that are not acid-soil specialized, but have mechanisms to exclude or otherwise deal with the relatively low levels of Al^3+^ encountered under moderately acid soil conditions (Al excluders). On one hand we might expect that *Anthoxanthum* responds to Al in a manner similar to domesticated grasses because there are similarities in the Al stress response even between some distantly related plants [16,22]. For example, *Arabidopsis* and grasses both release chelating organic acids at the root tip which prevent Al uptake [23]. It has been argued that release of organic acids in particular provides a ubiquitous and metabolically inexpensive avenue for the evolution of Al resistance across the plant kingdom [16]. On the other hand, Al-tolerance is unusually high in *Anthoxanthum* and thus qualitatively different from closely related cereals. For example, 160 uM Al exposure is sufficient to reduce average root growth in rice seedlings to 60% of normal, and root growth of maize, wheat and sorghum to less than 10% [24]. However, it takes upwards of 300 uM Al^3+^ activity to reduce root growth in *Anthoxanthum* seedlings by the same relative amount [3,25]. *Anthoxanthum* tolerance is more similar to that of Al accumulating plants although no accumulating species have been definitively identified among the grasses. The mechanism of Al uptake and vacuolar sequestration in accumulators is thought to involve different transport proteins than those used by excluders, and involves a wider range of organic acid ligands [15,26]. For this reason we might predict Al-tolerance in *A*. *odoratum* involves novel Al tolerance mechanisms and genetic pathways compared with domesticated grasses.

Our goals in the present study were to test whether *Anthoxanthum* is an Al accumulating or excluding species, to detect and functionally categorize genes with Al-responsive regulation, and to identify gene candidates for continued study. We examined the Al contents of *A*. *odoratum* tissues across genotypes of different tolerance levels and conducted deep RNA sequencing (RNA-Seq) of root-tips. We characterized the putative functions of transcripts based on genetic information from model plants including several cultivated grasses. We compare our results with what is known about the genes involved in Al tolerance in domesticated grasses. Finally, by comparing expression patterns between sensitive and tolerant *Anthoxanthum* genotypes, we highlight candidate genes of further interest in the ecological genetic study of rapid evolution at the PGE.

## Materials and Methods

### Plant material

*Anthoxanthum* inflorescences (seed families) were collected in July 2010 from the Park Grass Experiment (PGE, Rothamsted Research, Harpenden, UK). Special thanks to Dr. John Storkey, Dr. Andy McDonald for access to plant and soil samples. The Rothamsted Long-term Experiments National Capability (LTE-NCG) is supported by the UK Biotechnology and Biological Sciences Research Council and the Lawes Agricultural Trust. We collected seed from the most and least acidic subplots at the PGE so as to capture a selection of grass genotypes with a wide range of Al tolerance levels. The soil pH on the sampled subplots varies from pH 7.2 to 3.7 and extractable Al content varies from 0 uM (undetectable) on the highest pH plot to 836 uM on the most acid plot (extracted in 0.1 M CaCl_2_, see Table A in S1 File).

The Al tolerance of 108 plants from the PGE, including the genotypes used here for RNA-sequencing and qPCR, was tested using a hydroponic assay described in [27] (see also Fig A in S1 File). Briefly, seeds were planted in vermiculite, vernalized for 3 days and then germinated in a growth chamber. At 5 weeks, one seedling per seed family was transplanted to the greenhouse into a mixture of 1:1 perlite and vermiculite (8–16 plants per field subplot, avg. = 13.6). They grew in the greenhouse for a period of 10 months before tillers were removed for Al tolerance testing. Al tolerance was measured as relative root growth (RRG) of replicate vegetative tillers removed from each greenhouse plant. Root growth is a commonly used measure of potential fitness (yield) in cultivated grasses in response to acid or metal exposure (e.g. [28]), and it is the most commonly used method for quantifying metal tolerances in plants [29]. Tillers were removed and growth of newly initiated roots was measured in a low ionic strength hydroponic solution at pH 4. Relative root growth (RRG) was quantified as the difference in root elongation between an initial period of 4 days growing in the absence of Al versus a second phase growing 4 days with the added Al. Al was added to a final concentration of 950 uM, equivalent to 300 uM Al^3+^ activity in solution (calculated using [30]). An Al sensitive tiller is one that shows a large reduction in root growth during the second relative to the first phase of growth (negative RRG), whereas a tolerant tiller shows little reduction in growth upon exposure to Al (RRG ≈ 0).

### Al and nutrient contents of roots and shoots

Seedlings were grown from seed collected on the subplots listed above, plus two additional subplots, 10A/D (from which adult tillers were not tested). Sixty-four seedlings (5–8 seedlings per plot) were germinated in hydroponic solution solidified with 1% agarose and then transferred to liquid hydroponic solution where they were allowed to grow over the course of 21 days to generate a large number of roots. At the end of the growth period, RRG of each seedling was measured upon Al exposure as for tillers (above). At the end of the 4 day Al treatment period, approximately 1 cm long root tips were collected and frozen in liquid nitrogen. To generate enough tissue volume for measuring Al and nutrient content, tips were bulked into 11 pools according to seedling %RRG. (Here %RRG = 'root growth with Al' divided by 'growth without Al', was used rather than absolute RRG to standardize across seedlings of different sizes). Each pool contained root tips from 4–6 seedlings, the first sample from the six most Al sensitive seedlings (lowest %RRG), the second sample from the next six, etc. The remaining shoot and root tissue from the seedlings was pressed and dried in an oven at 40 deg C for approximately 48 hours. Dried tissue (29–33 mg) from the base of one mature leaf was cut from each dried plant and samples were bulked into 11 pools in the same manner as the root tips. The Al and nutrient content of the shoot samples was analyzed by inductively-coupled plasma spectrophotometry (ICP) following acid digestion. The root tissue was separated by centrifugation into two fractions for ICP analysis, the cell wall fraction and the symplast (internal cell contents, mostly vacuolar sap), following the method of [31]. One root tip sample was inadvertently lost during processing, so data on only 10 pools for both roots and shoots are reported here.

### RNA sequencing

From among 108 plant genotypes we selected for RNA sequencing one highly tolerant and one highly sensitive individual typical of the extremes of tolerance variation found at the PGE. The sensitive genotype was in the 6^th^ percentile for Al tolerance and the tolerant genotype was in the 96^th^ percentile for tolerance (Fig A in S1 File). We chose plants from soils at the PGE that receive the same fertilizer treatment but different lime treatments (subplots 4A and 4D) because they are most likely to have genetic diversity at Al tolerance-related loci while retaining some homogeneity of genetic background. Plot 4A is currently at pH 6.9 with extractable Al content below the ICP detection limit of 0.03 uM. Plot 4D is at pH 3.7 with extractable Al of 627 uM.

For RNA extraction, nineteen tillers from each genotype were separated from greenhouse plants and placed evenly into two hydroponic tubs. They were grown without Al for 9 days and then on day 10 the solution in one tub was replaced with Al-free solution (control treatment), the other with solution containing 950 uM Al. After 24 hours, approximately 1 cm long root tips were collected from all tillers and frozen in liquid nitrogen. Exposure of this duration was chosen in order to capture gene expression specific to Al exposure and not associated with secondary types of damage. To get enough RNA for sequencing, tissue was bulked into 4 groups, one control and one Al-treated sample for each of the two genotypes. Total RNA was extracted from each sample using Trizol (Invitrogen, Carlsbad, CA) followed by DNAse digestion. RNA quality was verified on a BioAnalyzer (Agilent) prior to preparation of one RNA-seq library for each sample (Table B in S1 File), followed by sequencing, was performed at the Weill Cornell Medical College using Illumina TruSeq paired-end sample preparation reagents and protocols. Each library was individually barcoded and sequenced in parallel on the Illumina HiSeq2000 platform using 100 bp paired-end reads.

### Transcriptome assembly

Initial output sequencing clusters were de-multiplexed and filtered using Illumina pre-processing standards. We further filtered the raw data by eliminating reads from all flow cell sectors with average base quality scores below 24 for any base position. The first 24 million reads from each of the four libraries were used to construct a composite *de novo* reference transcriptome using the assembler Trinity (version 2011-08-20) [32] using default parameters and a minimum contig length of 350 bp. The initial output contained 109,284 contigs. The assembly was then corrected for contig splitting and lumping errors using the error correction module of iAssembler v1.3 [33] with parameters that combine contigs with greater than 98% sequence similarity, at least 40 bp overlap, allowing 20 bp overhangs (input options—h 40 -e 20 -p 98). Correction condensed the initial contig set down to 88,103 unigenes.

### Functional annotation

All unigenes were subjected to protein homology comparisons and gene ontology (GO) term mapping using the suite Blast2Go [34]. Unigene sequences were compared with the GenBank non-redundant (nr) protein database using blastx with an E-value cutoff of 10^−6^. About 1.8% of sequences had highest scoring matches to microorganisms and were eliminated from further analysis. We also eliminated unigenes with no uniquely aligning reads, leaving 83,705 unigenes in the final root-tip transcriptome. Most unigenes in the set are likely to be transcribed from separate genomic loci, however some also represent divergent alleles or homeologs at the same genomic locus. A minority may also be unconnected fragments of the same transcript (see the use of iAssembler above). Hierarchical clustering alignments showed that some unigenes represent sets of alternative splice forms or transcripts with alternative stop and start sites (data not shown). These are indicated in the text and tables by unigene numbers with ranges (i.e. UN57026-8) and are represented separately in the tables. As with any *de novo* RNA-seq expression analysis, higher variance and less accuracy is expected for expression measurements of transcript isoforms, homologs, and homeologs with high sequence similarity, but this issue may be exacerbated in the analysis of polyploids. For this reason we have conducted corrections using iAssembler (above) and chosen a high expression cut-off for gauging significant up- and down-regulation (described below). The *Anthoxanthum* transcriptome assembly has been deposited in the GenBank TSA archive under accession number GBIE00000000.

Functional descriptors for each unigene were generated using the BLAST Description Annotator (BDA) in Blast2GO using default significance cutoffs. We define 'known' *A*. *odoratum* unigenes as those with any type of functional terms in their associated BDA descriptions. We define 'unknown' unigenes as those with no BDA description (no statistically significant descriptors detected) and those with BDA descriptions that contain no functional information (e.g. 'hypothetical protein'). The latter are mostly matches to predicted open reading frames within sequenced plant genomes. The BDA description provides a good hypothesis as to the general function of each unigene, but functional testing will be needed to confirm these predictions. We assigned GO terms to the known unigenes using confidence level cutoffs based on the default Blast2GO annotation configuration and evidence code weights [34]. The resulting GO terms were further generalized into the set of terms relevant for plant taxa using the reduced GO-Slim plant set (http://www.geneontology.org/). We also conducted BLAST protein homology based searches for 15 specific Al tolerance candidate loci from other species within the root tip transcriptome using stand alone BLAST (tblastn algorithm) [35].

### Differential expression

To measure unigene expression, reads were aligned to the transcriptome using TopHat v.1.3.2 [36]with default parameters (see http://tophat.cbcb.umd.edu/manual.shtml) using the aligner Bowtie and a predicted read-pair separation of 40 bp. The program DESeq [37] was used for calculating foldchanges in expression between the control and Al treatments for each genotype. Expression values are reported as basemeans which are normalized by library size and unigene length [38]. Unigenes that are up-regulated have expression foldchanges >1.0; those that are down-regulated have expression foldchanges <1.0. The significance of foldchanges in expression for each unigene, between treatments and between genotypes, was calculated using DESeq which employs an adjusted p-value (padj) calculation that controls for multiple testing [37]. To identify potential candidate genes for further study, we used the distribution of expression variation across all 83,705 unigenes between treatments (within genotypes) as an estimate of the distribution of variation between biological replicates within genotypes [37]. This method provides an estimate of variance within genotypes in targeted experiments such as this where the vast majority of all unigenes across the transcriptome are not affected by the treatment.

We identified candidate genes involved in the response to Al stress as those that are strongly up- or down-regulated in response to Al in both sensitive and tolerant plant genotypes (using an adjusted p-value cut off of 0.05). The ‘Al-response unigenes’ fall into categories +/+ and-/- (tolerant expression /sensitive expression) in Table 1. We calculated the difference between foldchanges in the tolerant and sensitive genotypes (considering foldchange values with padj>0.05 equivalent to zero) for all unigenes. Unigenes with foldchange expression between the tolerant and sensitive genotypes was ≥ 4 or ≤ -4 we consider for further investigation at the PGE as potential targets of selection in the evolution of Al tolerance.

**Table 1:** Al responsive unigenes by RNA-seq expression pattern.

			sensitive	
		(+)	(NS)	(-)
	(+)	92	263	0
		(51/41)	(153/110)	(0/0)
tolerant	(NS)	83	82,250	36
		(36/47)	(32,488 / 49,762)	(20/16)
	(-)	10	936	35
		(5/5)	(458/478)	(16/19)
			sensitive	
		(+)	(NS)	(-)
	(+)	92	263	0
		(51/41)	(153/110)	(0/0)
tolerant	(NS)	83	82,250	36
		(36/47)	(32,488 / 49,762)	(20/16)
	(-)	10	936	35
		(5/5)	(458/478)	(16/19)

Up (+) and down (-) unigenes have large expression differences between the control and Al treatments; NS, expression change between treatments is small (padj>0.05, see methods). In parentheses the number of unigenes with a described function is followed by the number with unknown function.

### Quantitative PCR

Twenty-two unigenes were selected for quantitative (qPCR) validation. We included unigenes with both high and low expression levels (primers listed in Table C in S1 File). Expression was measured in control and Al treated root tips from 7 PGE plant genotypes that ranged widely in Al tolerance (Fig A in S1 File). Three to five replicate tillers from each genotype were grown hydroponically with and without Al, root tip tissue was collected, and RNA extracted as described above for the RNA-seq plants. Root tip collection was made 24 hours after exposure to Al. cDNA libraries were prepared from each sample using iScript cDNA synthesis (BioRad). We designed qPCR primers to amplify a portion of each unigene sequence (ABI Primer Express 3.0). Primers were 15–30 bp in length, designed to have similar melting temperatures (~60 deg C), and amplify 50–100 sequence fragments for each target (Table C in S1 File). When similar transcripts were detected in the assembly, we manually designed primers specific to each sequence to avoid amplifying more than one transcript with the same primers. In some cases we detected groups of related alternative splice forms based on Trinity clustering and hierarchical clustering alignments (MultAlign, [39]) and designed primers to detect only the form(s) that showed evidence of differential regulation in the RNA-seq dataset. qPCR analysis was performed in 384-well plates using SYBR Green reagents on the ABI platform. *Histone-3* was used as a control gene for the assay and expression was quantified using the—ddCt method [40] with an average of 3.0 replicates (range 2–5) per unigene sequence, per genotype, per treatment. We calculated linear regressions for qPCR expression value vs. Al tolerance across the 7 genotypes (Table D in S1 File).

## Results

### Al accumulates primarily in root cell walls and is correlated with tolerance

**Fig 1:**
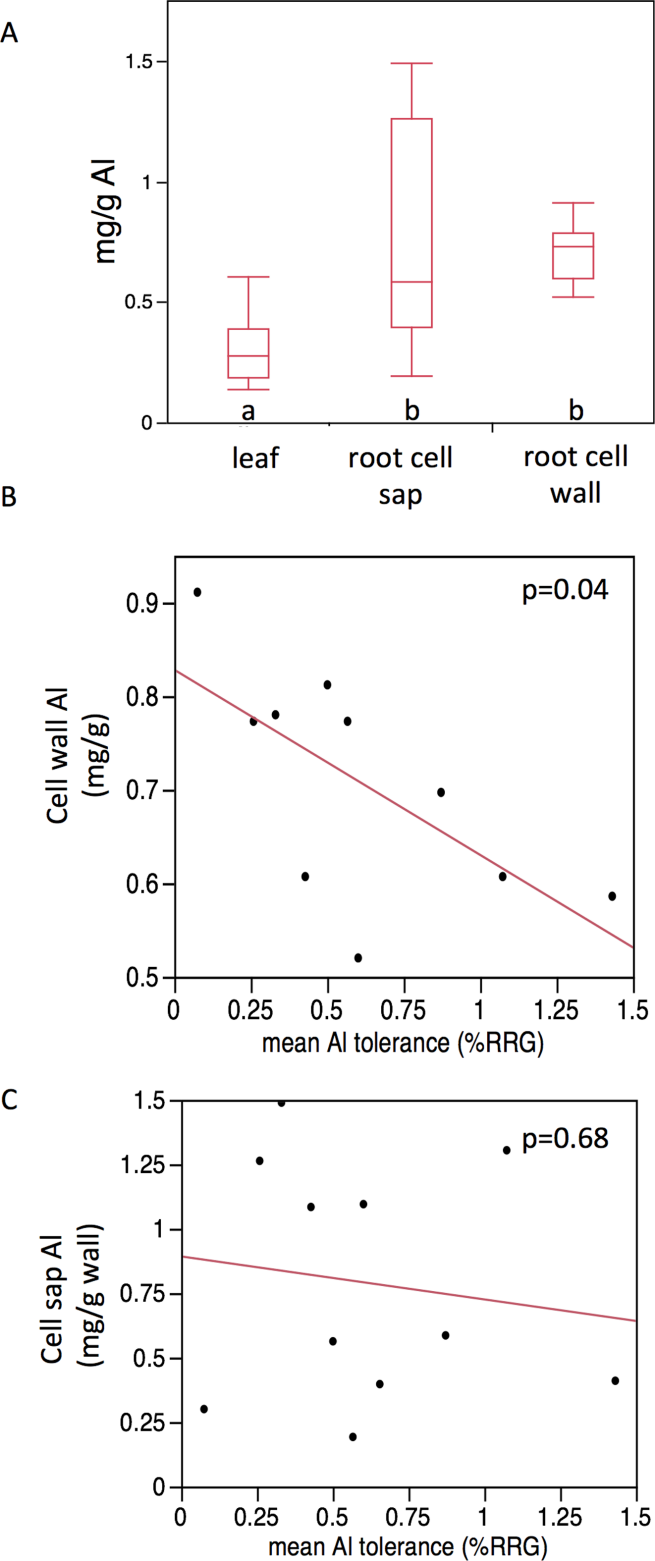
Al content of plants. A) three seedling tissues; B) and C) correlation of average tissue Al content of 10 seedling groups (see methods) with average group Al tolerance. (To control for seedling size differences, the average percent relative root growth values are used.) Cell sap Al content was standardized by mg cell wall in the sample. ns, non-significant. Levels with different letters are significantly different from each other at p<0.05 (Tukey’s HSD test).

In Al exposed seedlings, Al was clearly partitioned between plant tissues (F(2,29) = 8.34, p = 0.001, Fig 1A). Leaf tissue on average had 44% as much Al as root tip cell walls and 40% as much Al as the root tip cell sap. The Al contents of roots and shoots were not significantly correlated with each other (Table E in S1 File). Within the roots, lower Al content in the cell wall was associated with higher tolerance (Fig 1B, N = 10, r = -0.65, p = 0.04). There was no strong correlation between Al content inside the root cells (cell sap) and Al tolerance (Fig 1C, N = 10, r = -0.14, p = 0.68).

### Characteristics of the *Anthoxanthum* root tip transcriptome

Following sequencing and assembly, the final *de novo* root tip transcriptome contained 83,705 unigenes reconstructed from across the tetraploid genomes of two individuals (Table B in S1 File). The overall assembly had high coverage per unigene (average of 15 fragments per kilobase per million library reads, Fig B in S1 File). The mean unigene length was 803 bp, and fragmentation was low with half of all base pairs incorporated into unigenes 798 bp in length or greater. Most unigenes in the set are likely to be transcribed from separate genomic loci, however some also represent divergent alleles or homeologs at the same genomic locus. A minority may also be unconnected fragments of the same transcript (see the use of iAssembler in the Methods). Hierarchical clustering alignments showed that some unigenes represent sets of alternative splice forms or transcripts with alternative stop and start sites (data not shown). These are indicated in the text and tables by unigene numbers with ranges (i.e. UN57026-8).

We were able to describe almost half of the unigenes in the transcriptome based on significant BLAST matches to other species (Fig B in S1 File). Overall, the transcriptome had higher similarity to more closely related grasses: *Brachypodium*, followed by barley, wheat, sorghum, rice, and corn respectively (Table F in S1 File). Forty-seven percent of *Anthoxanthum* unigenes had significant similarity to previously described proteins (at E-value <10^−6^) and 32.8% of all unigenes could be assigned significant GO terms. The annotated unigenes with GO terms for molecular functionality span 17 diverse categories (Fig B in S1 File). Of note, a large proportion had nucleotide, DNA, or protein binding capacity, kinase activity and transporter activity.

### Early response to Al exposure involves a small set of transcripts

Response to Al exposure over 24 hours involved differential regulation of a small proportion of the overall transcriptome. Because of the short duration of Al exposure, the Al responsive transcripts we identified are more likely to be specific to Al exposure rather than induced by secondary stress such as dehydration or nutrient deficiency following root damage. In the presence of Al, 1,455 unigenes (1.7%, N = 83,705) were highly up- or down-regulated in one or both plant genotypes (Table 1A and Fig C in S1 File). To test the generality of gene expression measured by RNA-seq, we measured expression of a subset of 22 unigenes in a panel of seven PGE plant genotypes with a wide range of Al tolerance levels (Fig A in S1 File). We compared: wchange expression in the tolerant RNA-seq plant genotype with the average qPCR foldchange expression in four other tolerant genotypes, and expression in the sensitive RNA-seq genotype with three other sensitive genotypes (Fig A in S1 File). In both comparisons there was a significant positive relationship between expression change in the RNA-seq genotype and the average expression change measured via qPCR (Fig D in S1 File; tolerant plants, F(1,20) = 7.12, slope = 0.46 +/- 0.16, p = 0.013; sensitive plants, F(1,20) = 21.22, slope = 0.54 +/- 0.12, p<0.001). This shows that the RNA-seq data provides an accurate way of identifying transcripts that are differentially regulated in response to Al across genotypes with a wide range of Al tolerance levels. We also examined whether the difference in foldchanges between the Al sensitive and Al tolerant RNA-seq genotypes indicated an association between Al tolerance and transcript regulation across genotypes. Power to detect a statistically significant relationship was limited (n = 7 genotypes), and regression slopes for the relationship between gene expression change and Al tolerance were significant at p<0.10 for only 6 out of 22 unigenes. However the difference in expression between RNA-seq genotypes was predictive of the direction of correlation between expression change and Al tolerance 91% of the time (20 out of 22 unigenes, Table D in S1 File). If the relationship between expression change and Al tolerance were random, we would expect the prediction to be correct about 50% of the time. A 91% prediction rate indicates that differences in unigene regulation between the tolerant and sensitive RNA-seq plants are a reasonable predictor of how regulation corresponds with Al tolerance across genotypes at the PGE, however additional validation is warranted for future investigations of specific genes.

A set of 127 unigenes (0.01% of all transcripts) had a high level of up- or down- regulation in both RNA-seq genotypes (expression change in both genotypes was padj ≤ 0.05, categories +/+ and-/- in Table 2). Note, at this cut-off level, all of the identified unigenes were also up- or down-regulated by at least +/- 4-fold in one or both genotypes. About half had significant BLAST similarity to known loci and could be annotated with GO terms (n = 64). Of note, the set was enriched for extra-cellular region proteins and proteins involved in a wide range of metabolic processes including the metabolism of organic acids (Fig F in S1 File). Two Al-responsive malate transporters were up-regulated in both genotypes as was one glucosyltransferase and two udp-glucosyltransferases, which are involved in the transfer of sugars during cell-wall remodeling and growth [41,42]. Other Al-responsive transcripts of interest included two glutamate decarboxylases (involved in internal cytosolic pH regulation (Bouché & Fromm, 2004), several transfer RNAs and their synthetases, and a type of magnesium transporter (UN40932) known to be important in Al tolerance in rice [43]. Additionally, two transcription factors (UN02272 and UN02544/6), and a signaling kinase (UN06813) were up-regulated, which are putatively involved in the downstream activation of signaling cascades and gene expression following Al exposure. Some Al response unigenes also had putative functions not previously associated with Al tolerance in plants, e.g. a secologanin synthase (UN01958).

**Table 2:** Candidate unigenes for Al adaptation.

			sensitive	
		(+)	(NS)	(-)
	(+)	10	101	*NA*
		(7/3)	(63/38)	
Tolerant	(NS)	*NA*	*NA*	*NA*
	(-)	*NA*	234	0
			(139/95)	(0/0)

### Expression variation in both previously characterized and novel loci may be involved in rapid evolution at the PGE

We also identified a subset of unigenes that had a large difference in the degree of up- or down- regulation between the Al sensitive and tolerant genotypes (≥ 4 foldchange or ≤ -4 foldchange, n = 345, Table 2 and S2 File). The set includes some sequences that are Al response unigenes (differentially regulated in both sensitive and tolerant genotypes) but whose expression is much more responsive to Al exposure in one genotype. One hundred ninety-seven of the candidate transcripts could be described based on BLAST similarities and 134 could not. The annotated set was enriched for proteins localized to the extracellular region, but also those with transporter and hydrolase activity (Fig F in S1 File). Proteins involved in organic acid metabolism and aromatic compound metabolism were also over-represented. Among the unigenes that are more highly up-regulated in the tolerant plant were three putative malate transporters, two cation antiporters, an exonuclease-like transcript, three sugar transferases, several ATP-binding cassette (ABC) transporters, and an Nramp-type transporter.

For each expression category, the number of unigenes that are more Al responsive in the tolerant than sensitive genotype are listed. In parentheses the number of candidate unigenes with described function is followed by the number with unknown function. NA, not applicable. (see also S2 File)

We conducted direct BLAST searches of the transcriptome for the best protein matches (putative homologs) to 15 major Al tolerance genes that have been identified in other plants and directly examined their expression patterns. All but two of the *a priori* candidate loci (AtALT2 and AtBCB) had a high level of similarity to one or more *A*. *odoratum* unigenes (Table 3). It should be noted that multiple alignments showed the *Os*Nrat1 homolog is most likely represented by two un-joined fragments in the data set (UN08555 and UN37144). Six of the 15 candidates were up-regulated by at least two fold in response to Al including the putative homologs of *Ta*ALMT1, *Zm*MATE, *Os*Nrat1, *Os*ALS1 *Os*STAR1 and *Os*STAR2. All but two of these transcripts (*Zm*MATE and *Os*STAR2) were more Al responsive in the tolerant plant genotype (Table 3). *Zm*MATE, *Os*ALS1, and *Os*Nrat1 were included in the qPCR analysis (Table E in S1 File).

**Table 3:** Expression of putative homologs of Al candidate loci from other plant species.

				Tolerant (BaseMean)		Sensitive (BaseMean)		Tol.	Sen.
Gene(s)	Putative homolog	E-value	length (bp)	-Al	+Al	-Al	+Al	F.C.	F.C.
*OsALMT1*	UN14079	2.00E-128	1,120	794	8,882	626	3,351	11.2	5.4
*TaALMT1*		1.00E-157							
*OsFRDL4*	UN08191	6.00E-96	1,279	12,650	35,397	13,594	37,354	2.8	2.7
*ZmMATE*		2.00E-169							
*OsNrat1*	UN08555	3.00E-91	882	59,311	277,473	65,284	166,925	4.7	2.6
*OsSTAR1*	UN11920	2.00E-77	1,041	3,016	9,656	2,064	5,885	3.2	2.9
*OsSTAR2*	UN50094-6	2.00E-92	1,123	3,002	6,199	112	115	2.1	1.0
*AtALS3*		7.00E-85							
*OsALS1*	UN13071	0	2,194	29,867	70,247	24,741	39,888	2.4	1.6
*OsART1*	UN65068	1.00E-60	1,528	6,215	5,562	4,845	4,473	-1.1	-1.1
*OsMGT1*	UN33791	5.00E-64	1,821	9,355	13,719	9,887	14,720	1.5	1.5
*OsISL*	UN82594	2.00E-31	386	2	2	31	70	1	2.3
*AtATR*	UN01856	2.00E-174	692	789	1,145	1,021	931	1.5	-1.1
*AtALT2*	UN77886	2.00E-09	1,214	2,879	2,998	3,409	2,966	1	-1.1
*AtBCB*	UN84836	8.00E-19	409	1,041	1,565	483	983	1.5	2.0

Expression of putative homologs of Al candidate loci from other plant species in Al sensitive and Al tolerant *A*. *odoratum* genotypes.-Al, control treatment; +Al, Al treatment; BaseMean value = number of reads/ library geometric mean number of reads. E-values are from sequence comparison using tblastn.

## Discussion

Studies of adaptation to polluted soils have provided some of the most concrete examples of rapid evolution in plants [2,44–48]. Information on the genetic basis of edaphic tolerances continues to be generated from molecular work on domesticated plants and now provides a useful comparative resource for investigating the genetic basis of adaptation in natural populations. Short-read sequencing also now makes it feasible to search for and identify genes that are potentially associated with adaptive variation across the entire genome while at the same time examining *a priori* candidates. Studies of local adaptation to soil Al in the grass *Anthoxanthum* growing at the Park Grass Experiment were one of the early documented examples of rapid evolution in plants, and work on Al tolerance in cereal crops has now provided a way to leverage transcriptome-wide sequence information to identify candidates for the underlying genes. While early differential gene expression may not explain all the variation in Al tolerance found at the PGE, the RNA-seq approach allows a window into potential sources of genetic variation in non-model species. Our goals in this study were to identify genes involved in the early response to Al stress in *Anthoxanthum*, a highly tolerant wild grass, and to isolate a subset of loci for continued research on the genetic basis of rapid evolution at the site.

### *Anthoxanthum* has high Al tolerance despite lack of tissue sequestration

Results of tissue analysis show that although *Anthoxanthum’*s high level of Al tolerance is more typical of accumulating than excluding plants, the species is not likely to be an accumulator. Leaf concentrations of Al were low, well below the 1.1 mg/g threshold that has been suggested for detection of the trait in temperate species [49]. Al tolerance across individuals was not significantly correlated with the Al content of leaves or of the interior of root cells (Fig A and Table E in S1 File), but rather it was negatively correlated with Al content of cell walls. This suggests that Al exclusion via cell wall resistance to the attachment of Al (e.g. reduced interaction with pectin or other components, [50,51]) is more important for tolerance in this species than active Al uptake and storage.

### The genetic basis of the Al stress response

At the genetic level the Al stress early response in *Anthoxanthum* bears many similarities to the early stress response documented in other plants. It is difficult to directly compare transcriptional profiling studies of the Al stress response (which vary in the extent and duration of Al exposure), however generally our data indicate that the total number of Al-responsive loci in *A*. *odoratum* is within the same range as other plants: up to a few hundred loci [22,52–57]. The unigenes that respond to Al exposure in *Anthoxanthum* include all of the Al responsive functional types so far identified in studies of monocot and dicot plants. As categorized in *Arabidopsis* [54] they include genes involved in oxidative stress response, membrane transport, regulation of energy and metabolism, polysaccharide and cell wall metabolism, protein metabolism, signaling, hormones, and transcription factors. Interestingly, in *Anthoxanthum* there is also overlap between Al-response genes and loci putatively involved in early pathogen response, a pattern that has been documented in wheat but whose significance is not yet known [53]. For the large number of genes of unknown function involved in the Al stress response, we can say little except that they may facilitate or enhance tolerance conferred by the genes *Anthoxanthum* shares with other species. The unknown loci provide important targets for future research, particularly because they may underlie Al tolerance mechanisms not present in cultivated plants and may be linked to *Anthoxanthum*’s somewhat unique ability to thrive in Al toxic environments despite the apparent absence of Al tissue sequestration.

#### Organic acid transport genes

The most well-understood mechanism of Al-tolerance in plants is the exudation of organic acids at the root tip, which chelates Al^3+^ ions into a form that is non-toxic and not easily taken up by roots [58,16]. Chelation occurs in the zone just outside the root exterior, but may also be important in the root apoplast [13] or play a role in internal Al transport [15]. Members of two distinct gene families have been repeatedly linked to Al-responsive organic acid exudation in cultivated plants; the aluminum sensitive malate transporters (ALMTs) and the multidrug and toxic compound extrusion proteins (MATEs). In *Anthoxanthum* members of both families were up-regulated in response to Al, often more so in the tolerant plant genotype than the sensitive one. Two loci with close similarity to *Ta*ALMT1, the first gene linked to Al tolerance variation in wheat [59–61] were both up-regulated (UN14079 and UN11139, Table 4) suggesting that exudation or internal transport of malate plays a role in Al tolerance in *Anthoxanthum*. Malate exudation contributes to tolerance in several species, including *Arabidopsis* [23] and *Holcus lanatus*, another grass growing at the PGE [62]. Three separate MATE loci were also responsive to Al in *Anthoxanthum* (UN08191, Table 4; UN03495 and UN41821, Table D in S1 File), which suggests citrate transport plays a role in resistance to Al as well. Citrate exudation is the primary determinant of tolerance in sorghum [63], and is also important in barley [64] and maize [55, 65]. One of the three Al-regulated MATEs in *Anthoxanthum* (UN08191) is the probable homolog of the maize tolerance gene, *ZmMATE1*, while the other two bear little resemblance to this locus or each other. The presence of more than one Al-responsive *MATE* locus in *Anthoxanthum* contrasts with maize where among 45 MATE sequences only the *ZmMATE1* locus is Al responsive.. In general, the presence of multiple Al-responsive organic acid transport genes in both gene families in *Anthoxanthum* is somewhat unusual and has the potential to contribute to its exceptional tolerance. However, detailed quantification of organic acid content in roots and its relationship with Al sensitivity across genotypes will be required to test this idea.

**Table 4:** Al response unigenes in *A*. *odoratum*.

Unigene **(+/+)**	Description	length (bp)	Tolerant (BaseMean)		Sensitive (BaseMean)		Tol fold change	Sen fold change
			-Al	+Al	-Al	+Al		
UN38625	acetyltransferase-like protein	431	31	248	25	389	8.1	15.3
UN11139	almt1	715	148	2,014	297	1,456	13.6	4.9
UN14079	almt1	1,120	794	8,882	626	3,351	11.2	5.4
UN25744	anthranilate n-benzoyltransferase	629	168	1,526	34	373	9.1	10.9
UN00757	asparagine-trna ligase	428	1	560	4	202	ON	ON
UN17334	bifunctional aminoacyl-trna expressed	420	52	384	64	2,006	7.3	31.1
UN13369	cation h(+) antiporter 15-like	1,382	22	869	17	407	40.1	24.5
UN68709	cysteine synthase	841	128	871	45	497	6.8	11.1
UN68710		630	341	1,960	83	1,700	5.7	20.5
UN68711		619	343	2,013	81	1,877	5.9	23.2
UN27199	disease resistance response	745	373	1,108	29	471	3.0	16.1
UN12791	elongation factor 1-gamma	486	13	967	4	428	77.1	ON
UN26289	elongation factor 2	371	271	790	4	202	2.9	ON
UN04915	eukaryotic translation elongation	620	437	1,168	6	228	2.7	39
UN15889	eukaryotic translation initiation factor 6	538	128	1,928	94	2,116	15.1	22.6
UN40101	exonuclease-like protein	1,330	40	1,206	8	184	30.2	23.6
UN79476	flavonol sulfotransferase-like	1,269	302	3,277	132	1,981	10.8	15
UN23598	glucosyl transferase	961	78	695	20	875	9.0	42.7
UN23599		931	115	1,237	43	1,290	10.7	30
UN54955	glutamate decarboxylase	523	992	5,573	1,162	6,024	5.6	5.2
UN54956		418	2,487	12,784	2,373	12,157	5.1	5.1
UN67619	glutamate decarboxylase	590	449	1,984	260	2,058	4.4	7.9
UN67620		577	363	1,404	244	1,670	3.9	6.8
UN49474	histone-lysine n-methyltransferase atxr2-like	450	742	3,629	1,365	5,272	4.9	3.9
UN76040	indole-3-acetate beta-glucosyltransferase	678	285	795	183	1,475	2.8	8.0
UN10767	leucine rich repeat family expressed	548	891	4,754	965	4,734	5.3	4.9
UN10768		535	592	3,150	630	3,191	5.3	5.1
UN10769		499	468	2,543	517	2,504	5.4	4.8
UN10770		486	193	1,023	196	1,089	5.3	5.6
UN09895	lysyl-trna expressed	682	250	1,148	161	845	4.6	5.2
UN53468	lysyl-trna synthetase	369	234	1,389	155	1,131	5.9	7.3
UN40932	magnesium transporter mrs2-e-like	640	494	1,568	286	1,324	3.2	4.6
UN00039	map kinase	714	448	1,785	359	1,493	4.0	4.2
UN13967	methionyl-trna expressed	858	806	5,498	703	6,030	6.8	8.6
UN73493	r40c1 protein	484	75	804	172	1,542	10.7	9.0
UN38871	r40c1 protein—rice	584	503	5,657	253	3,280	11.3	13
UN48614	retrotransposon like protein	1,127	6	340	0	411	59.7	ON
UN52493	retrotransposon unclassified	519	1	99	0	111	ON	ON
UN01958	secologanin synthase-like	710	66	1,358	10	507	20.5	52
UN82177	threonyl-trna mitochondrial-like	376	7	311	5	328	45.5	ON
UN02272	transcription factor	1,273	316	1,377	239	2,572	4.4	10.8
UN12888	transferase family protein	696	124	898	27	317	7.2	11.6
UN10992	transmembrane expressed	1,295	838	2,907	384	1,674	3.5	4.4
UN21894	ubiquitin-60s ribosomal protein l40-like	424	9	341	9	273	37.4	31
UN10052	udp-glucosyltransferase	678	357	1,770	47	685	5.0	14.6
UN29927	udp-glycosyltransferase 74e1-like isoform 2	491	59	367	41	299	6.2	7.3
UN16805	valyl-trna expressed	529	466	1,774	127	998	3.8	7.9
UN16806		495	318	1,316	117	814	4.1	6.9
UN43368	valyl trna synthetase	1,697	3,691	18,104	508	4,832	4.9	9.5
UN68323	valyl-trna synthetase	673	1,112	5,737	243	2,467	5.2	10.2
UN27290	60s acidic ribosomal protein p2b-like	584	18	778	4	137	42.6	ON
41 (+/+) unigenes	*unknown function*							
Unigene **(-/-)**	Description	length (bp)	Tolerant (BaseMean)		Sensitive (BaseMean)		Tol fold change	Sen fold change
			-Al	+Al	-Al	+Al		
UN17541	ac115686_19 disease resistance response protein	1,103	2,478	378	2,044	152	-6.6	-13.4
UN54647	ankyrin-like protein	2,582	659	107	475	47	-6.2	-10.1
UN36964	bacterial-induced peroxidase precursor	383	206	36	145	7	-5.7	-20.7
UN66043	beta glucanase	634	1,220	109	895	76	-11.2	-11.8
UN19447	germin-like protein 8-9-like	1,026	1,276	12	140	2	-106.3	OFF
UN02725	nadph-dependent fmn reductase	579	2,168	62	149	0	-35.0	OFF
UN33751	papain-like cysteine proteinase	502	479	66	313	39	-7.3	-8.0
UN84680	pathogenesis-related maize seed protein	455	2,412	189	1,353	189	-12.8	-7.2
UN11563	pathogenesis-related protein 1	1,159	6,080	2,562	13,130	2,793	-2.4	-4.7
UN34450	precursor of carboxylase h-protein glycine decarboxylase complex	705	328	61	715	84	-5.4	-8.5
UN42833	regulator of ribonuclease activity a	370	1,420	257	2,629	264	-5.5	-10.0
UN04098	regulator of ribonuclease-like protein	456	505	105	2,448	315	-4.8	-7.8
UN02544	root abundant factor	614	315	35	360	50	-9.0	-7.2
UN06813	serine threonine-specific receptor protein kinase-like	3,011	1,657	612	1,320	303	-2.7	-4.4
UN18564	40s ribosomal protein s6	856	3,224	178	200	12	-18.1	-16.7
19 (-/-) unigenes	*unknown function*								

Al response unigenes in *A*. *odoratum*. Unigenes shown in the table have either significant up-regulation (+/+) or down-regulation (-/-) in both the tolerant and sensitive plant genotypes in response to Al exposure.-Al, control treatment; +Al, Al treatment; BaseMean value = number of reads/ library geometric mean number of reads. Where expression was indicated by a basemean of 5 or less, expression was considered zero in a treatment and foldchange is defined as simply “ON” or “OFF”

#### Internal Al detoxification genes

Transcript expression patterns also suggest that *Anthoxanthum* may internally detoxify Al^3+^ ions that enter the cell. Among grasses, internal detoxification via active transport and vacuolar storage has only been described in rice, the most tolerant of the cereal crops. In rice a plasma-membrane bound Nramp family Al transport protein, *Os*Nrat1, removes Al from the apoplast and a coordinated vacuolar ABC-transporter, *Os*ALS1, moves it into the vacuole [31,66]. Unigenes with strong similarity to OsNrat1 (fragments UN08554-5 and UN37144) and OsALS1 (UN13071) are up-regulated in response to Al in *Anthoxanthum* and also 10 to 100 times more highly expressed than the majority of other Al-responsive unigenes (Table 3).

#### Cell wall modification genes

A negative correlation between cell wall Al content and relative root growth (Al tolerance) across genotypes supports the idea that *Anthoxanthum* varies in its ability to prevent Al^3+^ from attaching to the cell wall and contributes to tolerance variation. We also found a suite of Al-sensitive loci in *Anthoxanthum* that bear strong similarity to genes involved in cell wall protection in rice. The putative homologs of two coordinated cell-wall modifiers, OsSTAR1 and OsSTAR2 (Table 3) are both moderately up-regulated in response to Al. In rice, these Al-induced, half-size ABC-transport proteins are localized to vesicle membranes and function in the release of UDP-glucose at the cell wall, which shields Al^3+^ binding sites [67]. Two udp-glucosyltransferases, a xyloglucan endotransglycosylase, and a cell-wall loosening enzyme from *Arabidopsis* [68] (Table 4 and S2 File) also all respond to Al exposure in *Anthoxanthum*.

#### Co-regulation

We cannot directly determine from our data the role of cis- versus trans- regulation in Al tolerance in *Anthoxanthum*, but it is notable that several of the candidate loci thus far described are regulated by a single master-regulator in rice, *Os*ART1 [69] and by a similar (but non-homologous) regulator *At*STOP1 in *Arabidopsis* [70]. The targets of *Os*ART1 include *Os*Nrat1, a UDP glucuronosyl/glucosyltranferase, *Os*ALS1, *Os*STAR1 and 2, a Mg transporter, cytochrome P450s, and a MATE locus, all of which are responsive to Al-exposure in *Anthoxanthum*. This supports the idea that a similar master regulator modulates the Al stress response in *Anthoxanthum*. On the other hand, there is no strong candidate for a homolog of OsART1 in the *Anthoxanthum* transcriptome: the unigene with greatest similarity to *Os*ART1 (UN65068) is only 33% similar and only 36% similar to the putative ART1 locus in its closer relative *Holcus lanatus*. Also, at least seven loci of unknown function are regulated by OsART1 in rice, but we did not find any Al-regulated unigenes similar to these in *Anthoxanthum*. For these reasons we find it more likely that if regulation of internal and external Al stress responses in *Anthoxanthum* is co-regulated, it is by transcription factor(s) that are not the C2H2 zinc-finger proteins implicated in co-regulation in other species. Possible candidates for such a regulatory locus include two Al-responsive transcription factors (UN02272 and UN02544/6, Table 4).

### Future study of rapid evolution of Al tolerance at the PGE

Examining differences in candidate gene regulation between representative tolerant and sensitive genotypes has revealed many loci of future interest in the study of the genetic basis of rapid evolution at the PGE. Some previously described Al response genes were more Al-responsive in the tolerant genotype including four ALMT-like loci (Table 4, UN14079 and UN11139; S2 File, UN11141 and UN47817). The same is true of the putative homologs of *Os*NRAT1 and to a lesser extent *Os*STAR1 and the three MATE loci (discussed above). In general, however, only about 60% of the transcripts with large differences in expression pattern between Al tolerant and sensitive individuals could be assigned a functional description (Table 2). For this reason, it is likely that *Anthoxanthum* employs tolerance mechanisms that are unique among non-cultivated grasses. Continued study of the *Anthoxanthum* candidate loci identified in the present work will be a valuable way to utilize the Park Grass Experiment for understanding how the origin of natural genetic variants, their genetic architecture, and the dynamics of selection upon them shapes the process of adaptation in wild plant populations.

## Supporting Information

S1 File Supporting Figs and Tables. (PDF)

S2 File Transcripts with potentially adaptive expression variation. Foldchanges under padj <0.05 (see methods) are listed as 'ns' and considered equivalent to zero foldchange for simplicity. Transcripts were considered potentially adaptive if the foldchange difference between tolerant and sensitive genotypes was > = 4.0 or < = -4. Expression values are given for all isoforms of the same locus where relevant. Where expression was indicated by fewer than 5 basemean reads in one treatment, foldchange is recorded as simply turned "ON" or "OFF".
(XLSX)
