# Supplementary material for: De Novo Transcriptome Assembly and Identification of Gene Candidates for Rapid Evolution of Soil Al Tolerance in Anthoxanthum odoratum at the Long-Term Park Grass Experiment
Source: PLoS One. 2015 Jul 6;10(7):e0124424. doi: 10.1371/journal.pone.0124424 (PMC4493143; doi:10.1371/journal.pone.0124424)
Supplement: S1 File — (PDF) [file pone.0124424.s001.pdf]

Figure A. Al Tolerance of plant genotypes from PGE. Grey points represent 107 plants measured for Al tolerance. Black points are genotypes analyzed using RNA-Seq; white points are genotypes used for qPCR analysis. RRG (relative root growth) values are LSMeans from a model that accounts for experimental block effects. Bars represent +/- 1S.E.

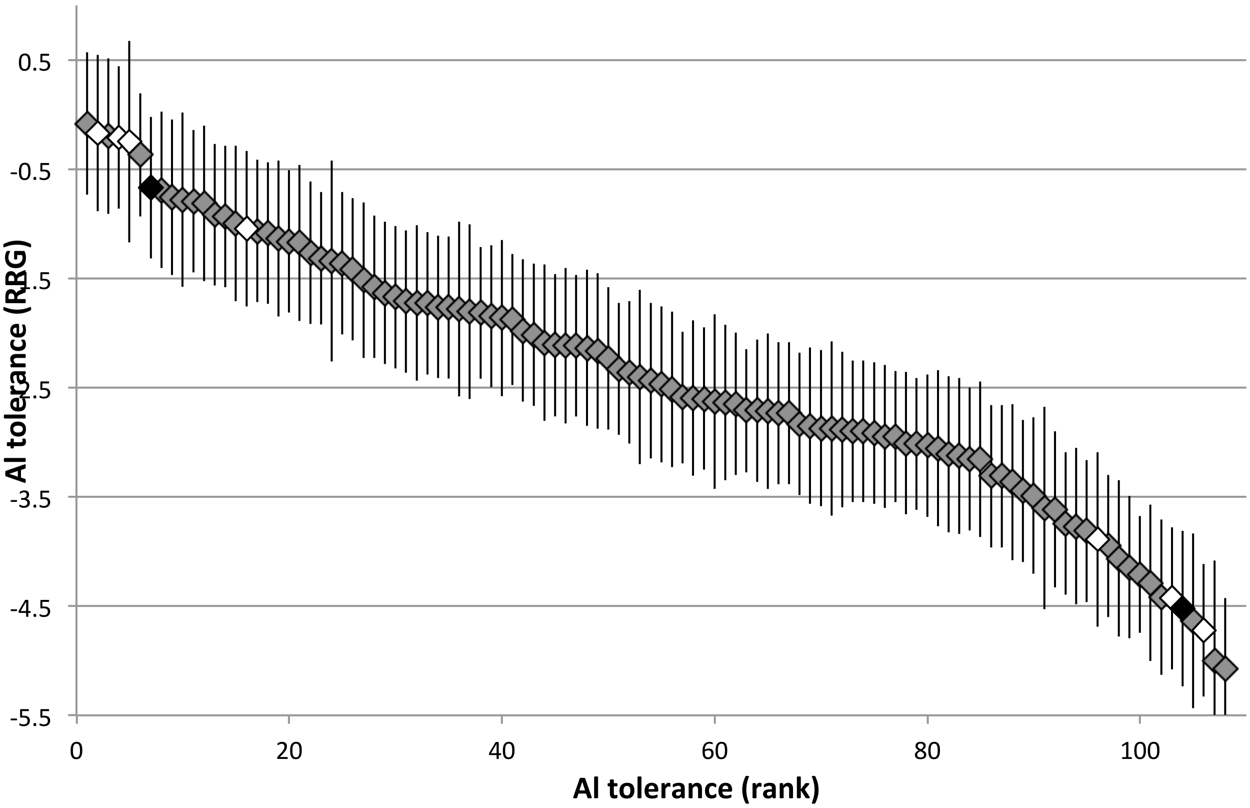

Figure B. Whole root tip transcriptome annotation statistics. A) Categories of annotated sequences with GO terms for molecular function. B) Species with the most Blast hits to *Anthoxanthum*. C) Number of sequences with significant Blast hits (e-value $<10^{-6}$) and GO annotations.

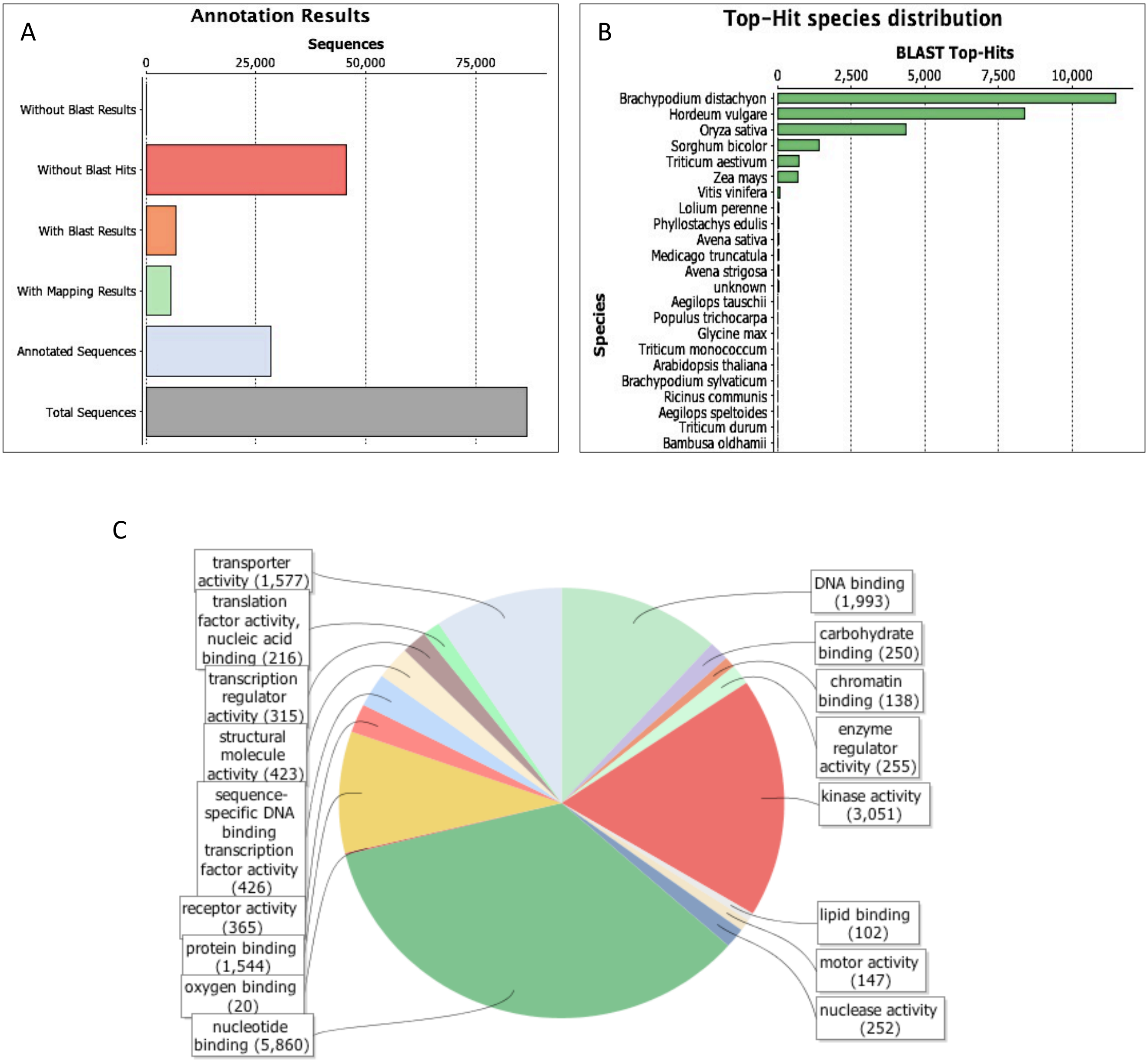

Figure C. Normalized (basemean) RNA-seq expression values in a) an Al tolerant and b) an Al sensitive plant genotype. Unigenes with zero expression values in the control or Al treatment are not shown (tolerant n=6.6% of unigenes, sensitive n=6.9%). Points in blue are significantly down-regulated in response to Al, and points in red are significantly up-regulated.

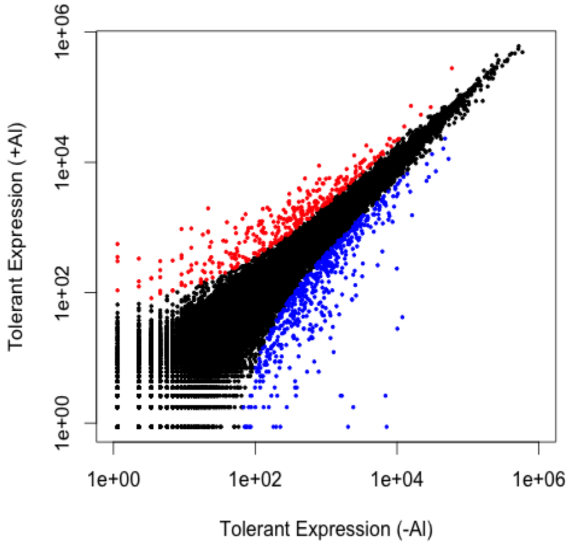

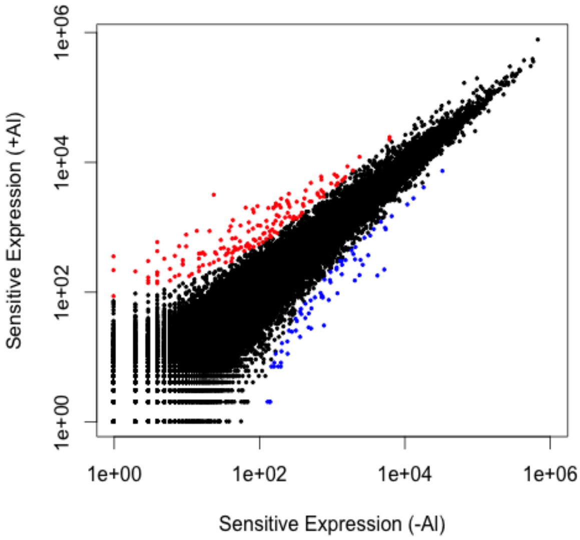

Figure D. **qPCR vs. RNA-seq expression change comparison.** Each point represents a separate unigene. A) qPCR average of three sensitive genotypes vs. the RNA-seq sensitive genotype. B) qPCR average of four tolerant genotypes vs. the RNA-seq tolerant genotype. Significance of the regressions was determined by ANOVA.

A

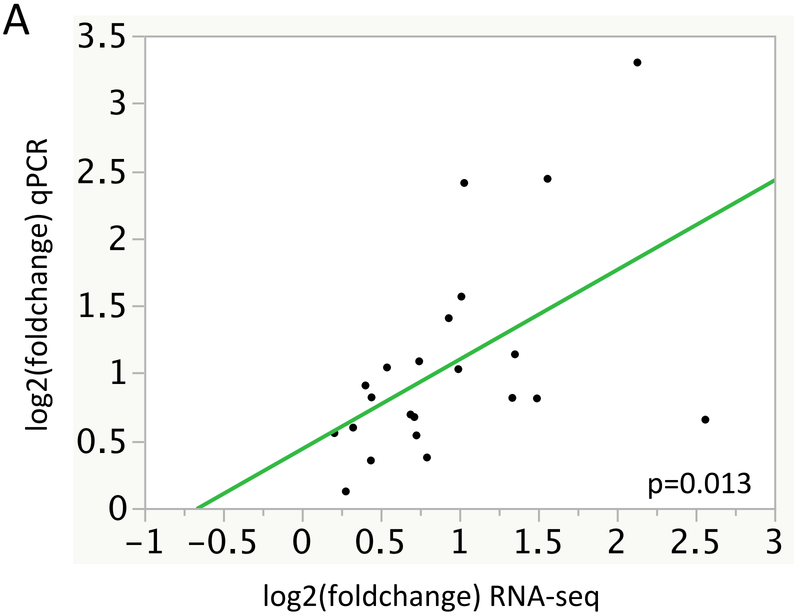

B

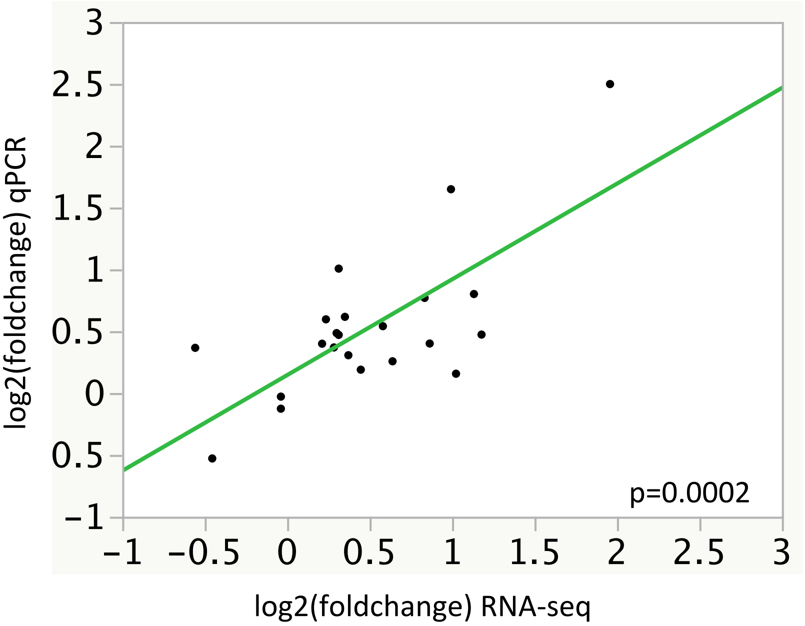

Figure E. RT-PCR graphs for 22 transcripts. Rq, relative quantıty of transcripts. Light grey bars, control treated roots; Dark grey bars, Al treated roots (see methods). Al tolerance of plant genotypes is listed by rank where 1 is the least tolerant and 7 is the most tolerant. Error bars represent +/- 1 stdev (note: in some cases bars are too small to be seen). * and * indicate that the expression difference between Al and control treatmented roots is significant up or down (at p<0.05, each), respectively. Upper right of each graph shows the RNA-seq foldchange in expression for the tolerant/sensitive genotype.

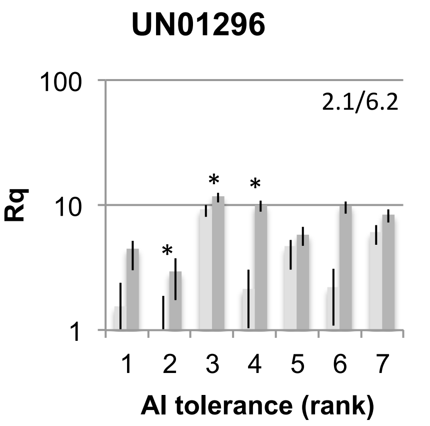

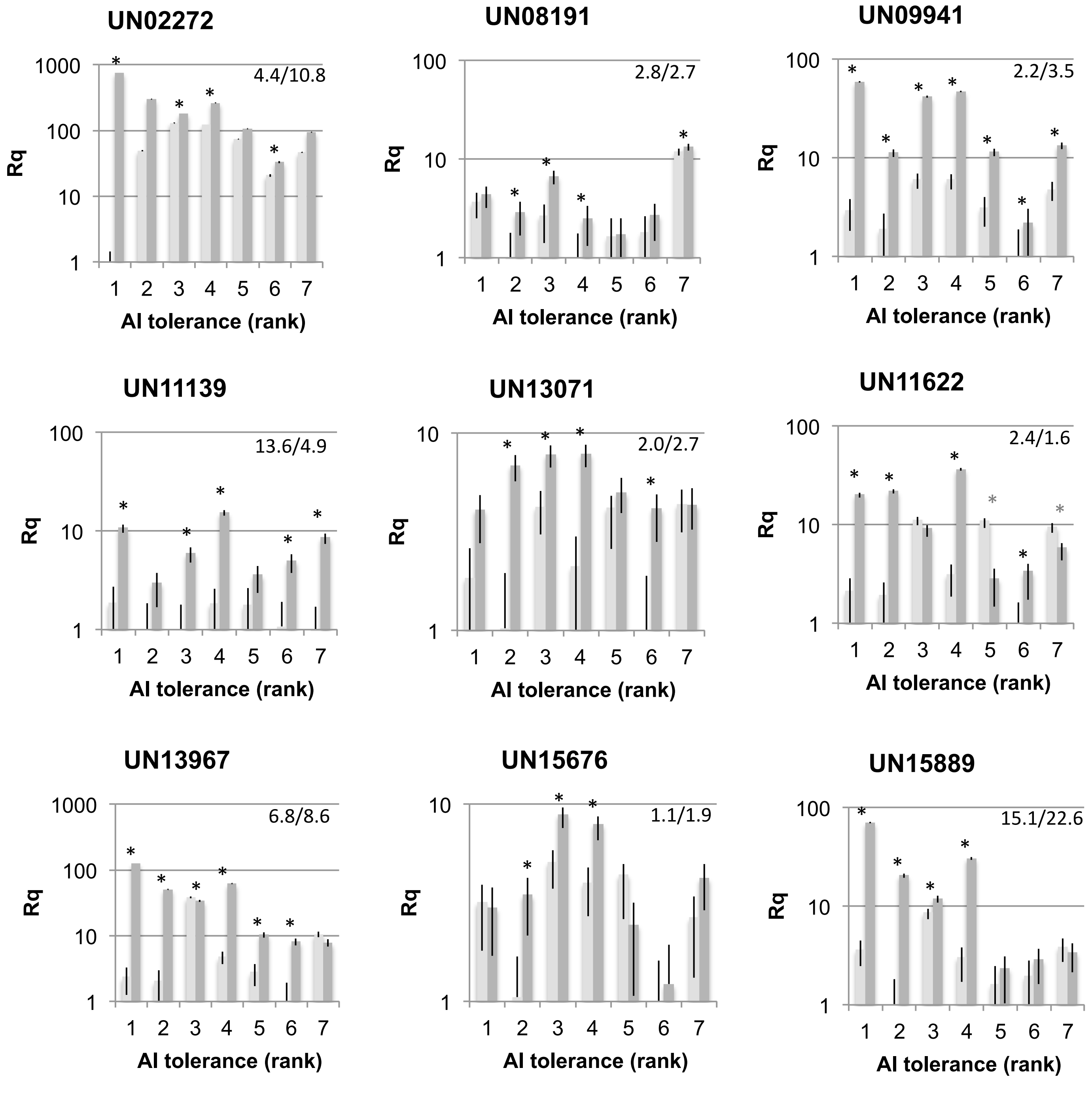

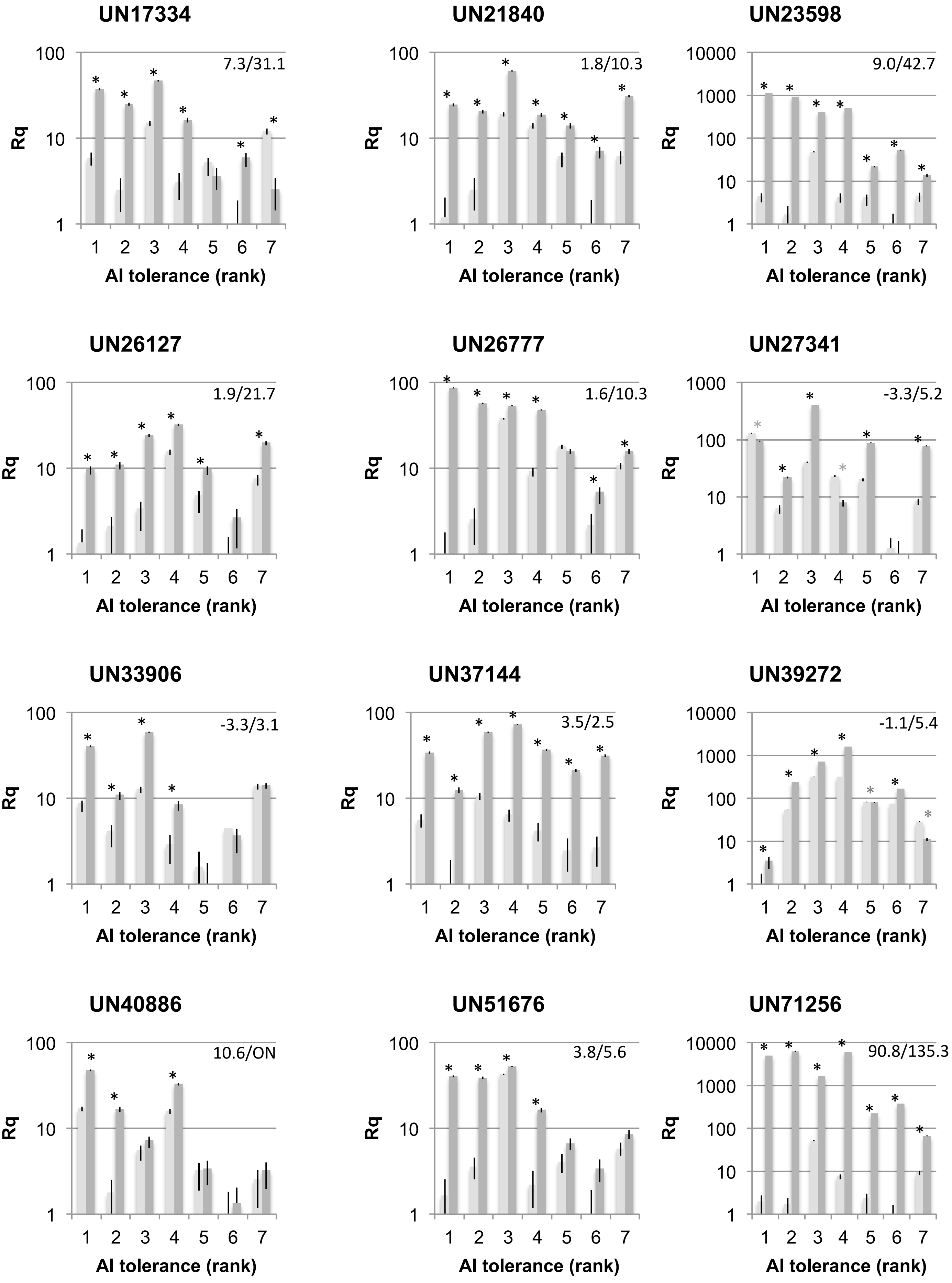
UN17334
7.3/31.1
UN21840
1.8/10.3
UN23598
9.0/42.7
UN26127
1.9/21.7
UN26777
1.6/10.3
UN27341
-3.3/5.2
UN33906
-3.3/3.1
UN37144
3.5/2.5
UN39272
-1.1/5.4
UN40886
10.6/ON
UN51676
3.8/5.6
UN71256
90.8/135.3
Rq
Al tolerance (rank)

Figure F. GO Term Enrichment Analysis. Terms overrepresented at $p<0.05$ are shown. A) Al response unigenes B) potentially adaptive candidate unigenes. Note, some GO terms are overlapping.

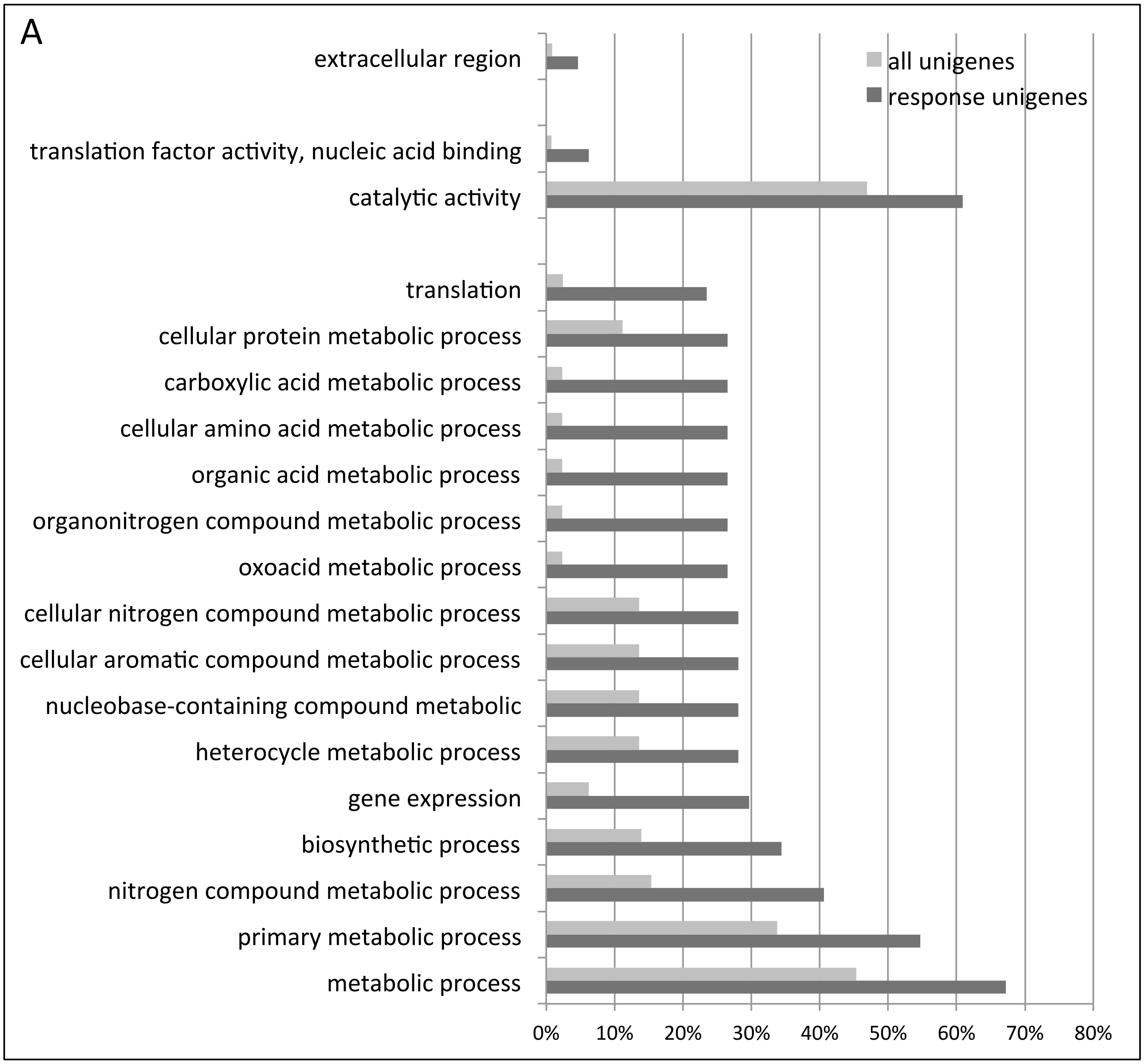

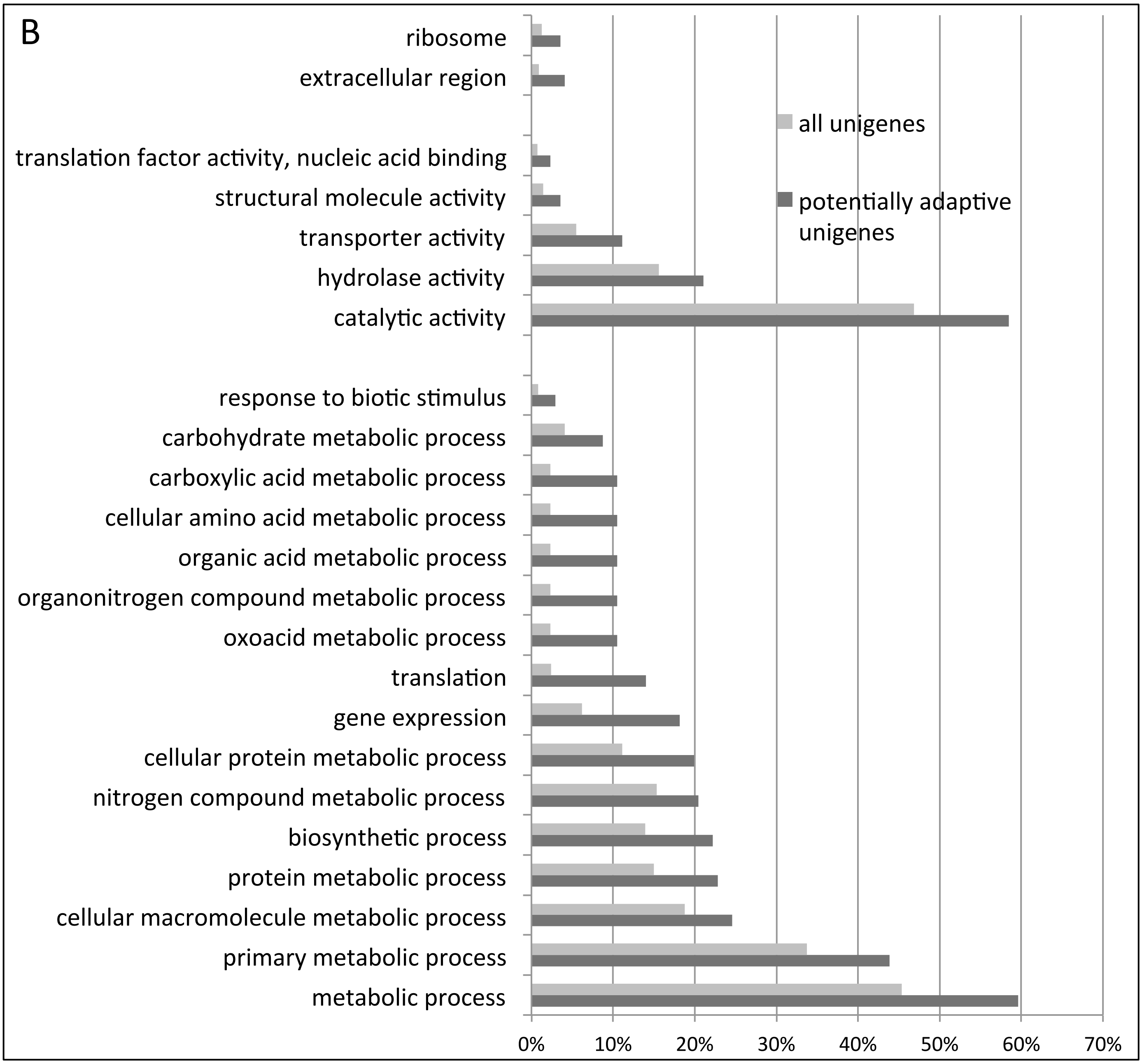
B
ribosome
extracellular region
translation factor activity, nucleic acid binding
structural molecule activity
transporter activity
hydrolase activity
catalytic activity
response to biotic stimulus
carbohydrate metabolic process
carboxylic acid metabolic process
cellular amino acid metabolic process
organic acid metabolic process
organonitrogen compound metabolic process
oxoacid metabolic process
translation
gene expression
cellular protein metabolic process
nitrogen compound metabolic process
biosynthetic process
protein metabolic process
cellular macromolecule metabolic process
primary metabolic process
metabolic process
all unigenes
potentially adaptive unigenes
0%
10%
20%
30%
40%
50%
60%
70%

Supplemental Tables

Table A. Soil plots sampled at PGE and their extractable Al content. The A subplots are limed to pH 7 and the D subplots are un-limed. Nutrient treatment information is taken from Silvertown et al. 2006. <det, below the detection limit.

Nutrient Plot	Subplot (soil type)	soil nutrient additions	Soil pH	Al *uM*
1	1A (low Al)<br>1D (high Al)	ammonium sulphate	7.1<br>4.1	<det<br>836
4/2	4A (low Al)<br>4D (high Al)	ammonium sulphate, plus P	6.9<br>3.7	<det<br>672
9/2	9B (low Al)<br>9D (high Al)	ammonium sulphate, plus P, Na, and Mg	6.3<br>3.7	3<br>710
10	10A (low Al)<br>10D (high Al)	ammonium sulphate, plus P, Na, and Mg	6.9<br>3.7	<det<br>685
3	3A (limed control)<br>3D (unlimed control)	none	7.2<br>5.2	2<br>6

Table B. RNA-Seq statistics. A) 4 libraries aligned to the combined *de novo* transcriptome. B) *De novo* transcriptome assembly statistics. RPKM, reads per kilobase per million library reads, FPKM, fragments per kilobase per million library reads

Library #	Genotype	Treatment	Total reads ($x10^6$)	# Reads filtered ($x10^6$)	Avg base quality score (PHRED)	% reads aligned to reference	% of reference Unigenes hit (>=10 RPK)	average unigene expression (RPKM)
1	sensitive	control	155.8	103.6	33.2	67%	91.2%	10.4
2	tolerant	control	152.3	128.3	33.4	84%	91.2%	10.3
3	sensitive	+Al	184.0	143.9	33.4	78%	91.1%	10.4
4	tolerant	+Al	210.5	106	33.4	50%	91.3%	10.3

Input reads	% reads assembled	# Unigenes	Average FPKM	Mean Unigene length	N50
96M	47%	83,705	15.0	803	798

Table C. Primer sequences used for qPCR analysis.

UNIGENE TARGET	FWD	REV	Tm (F/R, deg C)
histone3 (CONTROL)	CACATATCACAACGCACACC	GAATTATCTGTCGCTGCCATTG	61/62
UN11622	CAAGGCAGCACCCTTATTGC	CGCTGAACCATGTCCTTGTG	59/59
UN08191	TCGTCGACACCGCATTCA	GATACACCTACAGCTGCAAGCTCTA	59/58
UN26777	CCGTTTTCTGTCACATCTAGAAGTCA	TATCCTTCATAGAACCCAGCTTGTT	60/58
UN67620	GGAATTTGTAACCCAAAGTTTTGC	CACGGGCATTCAACTAGTAAAAATAG	59/58
UN71256	CTGCAGCAGGCCCTTGA	GCAGCCGGAGCAACAATG	58/60
UN26127	CAATGGTCATTCCTGAAATGGA	TCGGCTTGATGCTTGAGTCA	58/59
UN02272	CCGTGTTCGCGGAAGGT	TGGGTGTCTGATCATCTTCATCA	59/59
UN09941	AGCAGGCAAGAAAGGAAGTGTT	AGCCGACCCAAGTTCTCAAA	58/58
UN37144	CATTCCTGAGCAGCAGAGGTGC	CGGTGGTTGTTGTTGCTGG	60/60
UN13071	GCTGGTTTTCGCCACCAT	TATCAATTATCTTGCCGCCATACTT	58/59
UN27341	TGGGCTCCGATGCCG	CTGAGGAGCCACCGGGAT	60/60
UN13967	GAATCGGAGTCTTTGGCAATG	TGATGCCTCAGGACGATTACTAAG	58/58
UN10052	GGTGTGAGAGTGCAGAAGAGCG	GGTGTGAGAGTGCAGAAGAGCG	61/60
UN40886	CGTCCATCACCGTCAAGCT	CCGTTGGAGCGGAGGAT	58/58
UN15889	GGTGGAATTCGGCAGAAGAA	CGGCGGCACGAGGAT	59/59
UN17334	CGTCATCCCCAGTTTGGTCTT	CTGCGGTTCGCAATCCA	60/59
UN39272	AAAGTAATGTCACCACATCGTGTTC	TCGTGCTGTTCTTCATTTTTCTTG	58/59
UN23598	AGCCGGCCGCCATGT	ATCCAATTGATCTTCCTCGAACTG	60/59
UN11139	CCAGCTCGCGCACGAT	AAGACGAAGACGGTGGTAAGGA	59/59
UN34546	CCTCGTGCAGCTTCACCAT	GCCAGTGGTAGAAGGTGTGTTTG	58/60
UN03316	TTATGACTCCTCTGTCCCCCG	GCCATCGTTTCTGGCTGCT	60/60
UN15676	GTGAGGACTCGAGGTCGCC	AGCAGAGCCTGCGGCAG	59/60
UN01296	GATACAATCAAAGAAGAATGTAGGAAGAGA	GCTATCTCGGATGCAGTTGCA	59/60
UN21840	AAAACGTACAAGGAGATAGAGATTGGA	AACACGGGAAGCTCGATGAG	59/59
UN33906	GAAGCAGACAGTCCCGGAGA	CTTCGCACTGTTCGTCGC	59/58
UN51676	GGGACTCGATCGGAGATACG	CCGTCGTGGTAGTCCCAGTAA	58/59

Table D. Regression slopes and significance for qPCR transcript expression vs Al tolerance. Column 2 lists the predicted direction of correlation between foldchange expression and tolerance (RRG) based on regulation in the RNA-seq gentoypes. Columns 3 and 4 list the linear regression slope and ANOVA p-value of the relationship between expression foldchange and Al tolerance across 7 plant genotypes (Fig S1). Columns 5 and 6 list the same for relative expression level under Al exposure vs. Al tolerance. Positive numbers are in red, negative in blue. Significant p-values for the regression ANOVAs are in bold.

Unigene	RNA-seq foldchange difference	QPCR foldchange vs. Al tolerance (RRG)		QPCR expression (Rq) vs. Al tolerance (RRG)	
		slope	p-value	slope	p-value
UN01296	-4.2	-0.1	0.94	-2.5	0.49
UN02272	-6.4	-359.8	0.26	-410.3	0.09
UN08191 (MATE *hom.*)	0.1	-1.1	0.21	2.5	0.60
UN09941	-1.2	-10	0.10	30.7	0.18
UN11139 (ALMT1-like)	8.7	2	0.53	0.61	0.90
UN11622	-0.8	-6.6	0.33	12	0.38
UN13071 (ALS1)	0.7	-2.8	0.30	-1.7	0.37
UN13967	-1.8	-28.6	0.15	-72.9	0.08
UN15676	-3.0	-0.7	0.73	-2	0.53
UN15889	-7.5	-15.9	0.07	-37.4	0.13
UN17334	-23.8	-5.9	0.14	-39.6	**0.003**
UN21840-3	-8.1	-8.3	0.24	-19.7	0.29
UN23598-9	-26.5	-311.1	0.13	-965.7	**0.01**
UN26127	-19.8	-5.2	**0.004**	-0.64	0.96
UN26777	-8.7	-46.3	0.15	-63	**0.008**
UN27341	-8.8	-0.7	0.89	-145.2	0.34
UN33906-7	-5.0	-5	**0.03**	-37.1	0.08
UN08555 (NRAT *hom.*)	2.1	2.8	0.36	-0.29	0.99
UN39272	-6.4	-4.5	0.11	-80.7	0.91
UN40886	-354.8	-2288.2	0.09	-4353.2	0.13
UN51676	-1.8	-3.9	0.21	-23.7	0.21
UN71256	-44.4	-12.7	0.14	-45.9	**0.001**

Table E. Pearson correlations between Al tolerance, Al content in tissues and nutrient content in leaves of seedlings. N = 10 pooled tissue samples (see methods). Significant correlations ($p \leq 0.05$) are in bold, marginally significant values are in italic ($p \leq 0.10$) cw: root cell wall, sap: root cell symplast.

	Al tolerance (%RRG)	cell sap Al	cell wall Al	leaf Al	Ca	Mg	K	P	B	Mn	Fe	Cu	Mo
cell sap Al	-0.14												
cell wall Al	**-0.65**	-0.33											
leaf Al	0.22	-0.08	-0.44										
Ca	0.32	-0.38	-0.03	0.22									
Mg	0.40	-0.26	-0.16	0.39	0.93								
K	-0.02	-0.42	-0.19	**0.65**	0.00	0.08							
P	0.11	0.12	-0.46	0.36	0.42	*0.51*	0.40						
B	0.24	0.19	**-0.69**	0.24	0.13	0.06	0.30	*0.53*					
Mn	0.33	-0.14	0.03	0.13	**0.81**	**0.83**	-0.02	0.33	0.17				
Fe	-0.49	-0.28	*0.57*	-0.09	*0.56*	0.40	0.01	0.20	-0.02	0.37			
Cu	-0.13	-0.24	-0.04	0.28	0.46	*0.54*	0.48	**0.85**	0.19	0.29	0.48		
Mo	0.38	-0.05	-0.10	0.18	**0.69**	**0.83**	-0.12	0.24	-0.23	**0.79**	0.12	0.23	
Zn	-0.32	0.06	-0.06	**0.60**	0.47	0.50	0.35	**0.61**	0.34	0.34	0.48	**0.61**	0.20

Table F. *Anthoxanthum* sequence similarity to model plant species. Similarity is calculated as the number of top BLAST hits to a species divided by the number of sequences available in the nr database for that species.

Species	# sequences in nr database	# top BLAST hits in *Anthoxanthum*	Similarity (% available seqs hit)
Brachypodium distachyon (brome)	25,545	16,256	64%
Hordeum vulgare (barley)	34,685	11,876	34%
Triticum aestivum (bread wheat)	10,748	910	8%
Sorghum bicolor (sorghum)	71,876	2,452	3%
Oryza sativa (rice)	280,612	6,562	2%
Zea mays (corn)	108,085	915	1%
Arabidopsis thaliana (thale cress)	224,411	30	<0.001%

Table G. Transcripts with potentially adaptive expression variation. Foldchanges under padj <0.05 (see methods) are listed as 'ns' and considered equivalent to zero foldchange for simplicity. Transcripts were considered potentially adaptive if the foldchange difference between tolerant and sensitive genotypes was >=4.0 or <= -4. Expression values are given for all isoforms of the same locus where relevant. Where expression was indicated by fewer than 5 basemean reads in one treatment, foldchange is recorded as simply turned "ON" or "OFF".

See separate file.
